# Effective treatment with intravenous immunoglobulin for Henoch–Schönlein purpura with refractory gastrointestinal symptoms in an adolescent: A CARE-compliant case report

**DOI:** 10.1097/MD.0000000000040370

**Published:** 2024-11-01

**Authors:** Liji Chen, Cailing Zhong, Longxiu Fan, Ming Luo, Linkun Cai, Beiping Zhang, Haiyan Zhang

**Affiliations:** a The Second Clinical Medical College of Guangzhou University of Traditional Chinese Medicine, Guangzhou, China; b Department of Gastroenterology, The Second Affiliated Hospital of Guangzhou University of Traditional Chinese Medicine, Guangzhou, China.

**Keywords:** case report, corticosteroid, delayed diagnosis, gastrointestinal, Henoch–Schönlein purpura, intravenous immunoglobulins

## Abstract

**Rationale:**

This article presents a complex case of refractory Henoch–Schönlein purpura (HSP), initially manifesting with complex gastrointestinal (GI) symptoms, and discusses diagnostic and therapeutic challenges encountered. It aims to enhance understanding of the disease and provide evidence for the potential efficacy of intravenous immunoglobulin (IVIG) treatment in this condition.

**Patient concerns:**

A 16-year-old male patient presented with persistent abdominal pain, nausea, vomiting, and constipation for 8 days, leading to hospital admission.

**Diagnoses:**

Establishing a definitive diagnosis was challenging initially due to the absence of typical petechiae. However, the appearance of characteristic petechiae subsequently confirmed the diagnosis of HSP.

**Interventions:**

Initial treatment with methylprednisolone sodium succinate for 3 days failed to elicit improvement. Subsequently, IVIG was introduced as a combination therapy.

**Outcomes:**

Following the combined administration of IVIG, the patient experienced complete resolution of abdominal pain, petechiae, and arthralgia within 4 days.

**Lessons:**

This case highlights the importance of considering HSP in the differential diagnosis of patients with complex GI symptoms. Furthermore, it suggests that IVIG may be a valuable therapeutic option for HSP patients with refractory GI symptoms. High-quality comparative trials are needed to establish more definitive evidence for the effectiveness of IVIG and to develop specific treatment guidelines.

## 1. Introduction

Henoch–Schönlein purpura (HSP), also known as immunoglobulin A vasculitis,^[1]^ is a common systemic vasculitis in children, with an annual incidence of 3 to 55.9/100,000.^[2–4]^ HSP can involve the skin, joints, gastrointestinal (GI) tract, and kidneys, with typical symptoms including purpura, abdominal pain, and arthralgia. In most cases, the initial symptom is skin purpura, whereas abdominal pain is less frequently observed as the first sign.^[5–7]^ When GI manifestations occur without accompanying purpura, establishing a definitive diagnosis can be challenging, often leading to misdiagnosis. For patients with severe abdominal pain, corticosteroid (CS) therapy is a common treatment. However, some HSP patients with refractory GI symptoms do not respond even to CS. In such instances, intravenous immunoglobulin (IVIG) is considered as a second-line treatment option. Currently, there is no consensus or standardized guidelines on the use of IVIG for HSP, particularly in patients with complex GI manifestations. Herein, we present an atypical case of HSP, characterized by complex GI manifestations as the initial symptom. Initially, CS therapy was ineffective, but after the combined administration of IVIG, the patient’s abdominal pain, petechiae, and joint pain were completely alleviated. The case report has been structured according to the CARE checklist.

## 2. Case description

A 16-year-old male was admitted to the gastroenterology department with complaints of recurrent abdominal pain lasting for 8 days, accompanied by nausea, vomiting, and constipation. Three weeks prior, he had undergone a “subtotal resection of left thyroid cancer” for thyroid malignancy and was prescribed levothyroxine sodium tablets postoperatively. He had no history of other chronic diseases or malignancies. Physical examination revealed normal vital signs, a non-tender and soft abdomen without signs of peritonitis, and normal bowel sounds. The laboratory findings are shown in Table 1. Anti-CMV-IgG, liver and renal function, and electrolytes were all within normal limits. Tests for antineutrophil cytoplasmic antibodies, autoimmune antibodies, parasites, Epstein-Barr virus DNA, rotavirus and *Clostridioides difficile* were negative. Given the elevated inflammatory markers, gastroenteritis was suspected. Treatment was initiated with cefoperazone/sulbactam, ornidazole, and glycerin enema.

**Table 1:** Laboratory tests on the day of admission and petechiae appearance.

Laboratory indices	Admission	Purpura appearance	Norm
Inflammatory indicators			
WBC (×10^9^/L)	14.94	5.99	4.1–11
NEUT (×10^9^/L)	12.81	4.52	1.8–8.3
NEUT (%)	79.2	75.4	37.0–77.0
ESR (mm/h)	36	/	0–20
PCT (ng/mL)	0.09	0.11	0–0.05
hs-CRP (mg/L)	52.88	/	0–6
Coagulation indicators			
PT (s)	15.5	17.2	11.0–14.5
PT% (%)	74.0	61.0	70–130
APTT (s)	39.0	45.6	28–45
INR (R)	1.37	1.37	0.8–1.2
FIB (g/L)	4.18	4.35	2–4
FDP (mg/L)	21.87	7.42	0–5
DDi (mg/L FEU)	6.21	2.10	0–0.5
Immunological indicators			
IgM (g/L)	0.45	–	0.50–2.55
IgA (g/L)	3.91	–	0.67–3.14

APTT = activated partial thromboplastin time, DDi = D-Dimerl, ESR = erythrocyte sedimentation rate, FDP = fibrin degradation products, FIB = fibrinogen, hs-CRP = hypersensitive C-reactive protein, IgA = immunoglobulin A, IgM = immunoglobulin M, INR = international normalized ratio, NEUT = neutrophil, PCT = procalcitonin, PT% = PT percentage activity, PT = prothrombin time, WBC = white blood cell.

**Figure 1.**
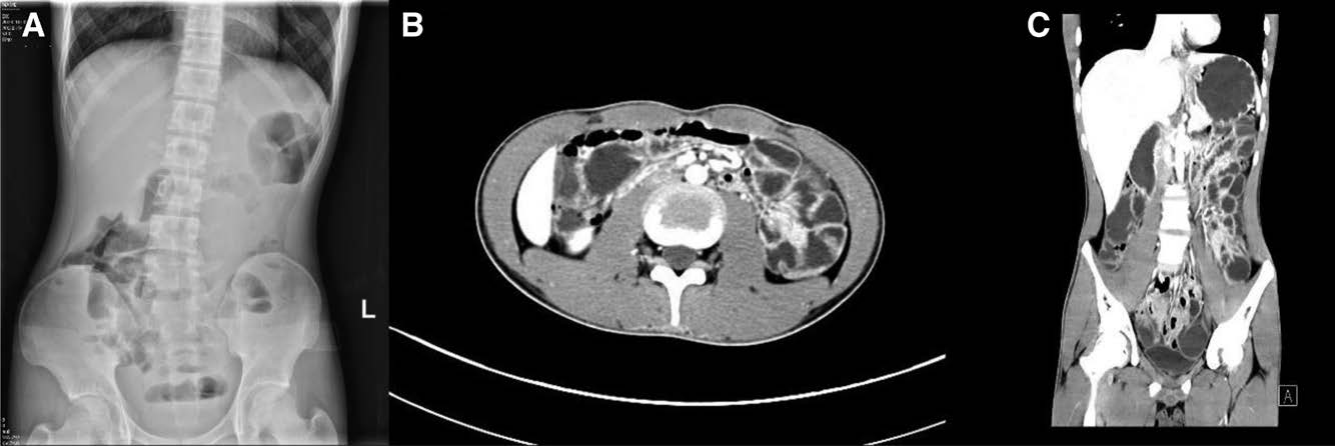
lmaging findings of the patient. (A) An erect abdominal X-ray showed intestinal distension and multiple air-fluid levels in the middle and lower abdominal intestines. (B and C) CTE of the small intestine showed a clustering in groups 3 and 4, as well as segmental mild thickening in group 3. CTE = computed tomography enterography.

On the 3rd day of admission, despite the normalization of the patient’s inflammatory indicators (Table 1), his abdominal pain and constipation unexpectedly worsened. An abdominal X-ray revealed dilatation and accumulation of gas in the middle and lower abdominal intestines, along with multiple short small air-fluid levels (Fig. 1A). Based on these findings, a diagnosis of incomplete small bowel obstruction was made. Subsequently, emergency treatment was implemented, including fasting, GI decompression, oral administration of lactulose and mosapride, glycerin enemas, and nutritional support.

**Figure 2.**
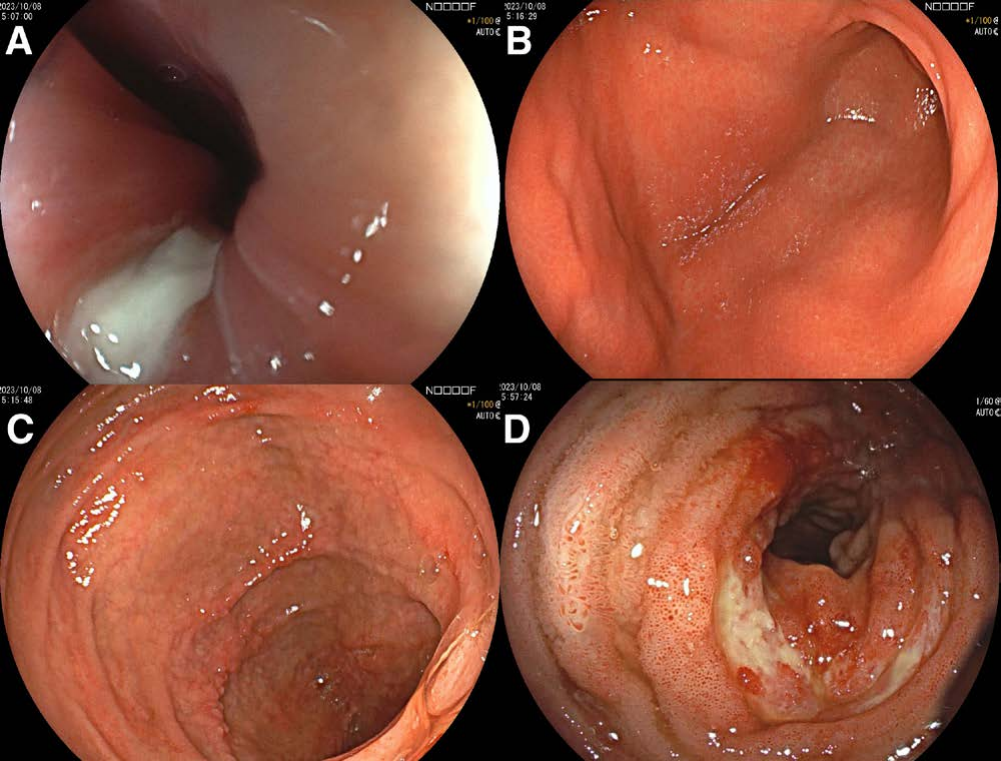
Endoscopic findings. (A) Multiple longitudinal ulcers with thick white moss in the esophagus. (B) Irregular ulcers with thin white moss in the duodenal bulb. (C) Multiple annular ulcers with pebble-like lesions in the surrounding mucosal layer in the descending portion. (D) Multiple circular ulcers with thick white moss, along with polypoid hyperplasia in the surrounding mucosa in the terminal ileum.

On the 7th day of admission, a repeat abdominal X-ray indicated the resolution of the intestinal obstruction, yet abdominal pain persisted. Subsequently, endoscopy revealed multiple ulcers in the esophagus, duodenum, and terminal ileum (Fig. 2). Specifically, the esophagus demonstrated multiple longitudinal ulcers covered with a thick white coating. The duodenal bulb exhibited irregular ulcers covered with a thin white coating, while the descending duodenum presented with multiple annular ulcers surrounded by pebble-like lesions. The terminal ileum displayed multiple annular ulcers with thick white coating, accompanied by polypoid hyperplasia in the adjacent mucosa. These findings led us to suspect a diagnosis of Crohn disease. Additionally, computed tomography enterography of the small intestine revealed clustering in groups 3 and 4, as well as a segmental mild thickening in group 3 (Fig. 1B and C). Pelvic MR enhancement examination did not reveal any abnormalities.

**Figure 3.**
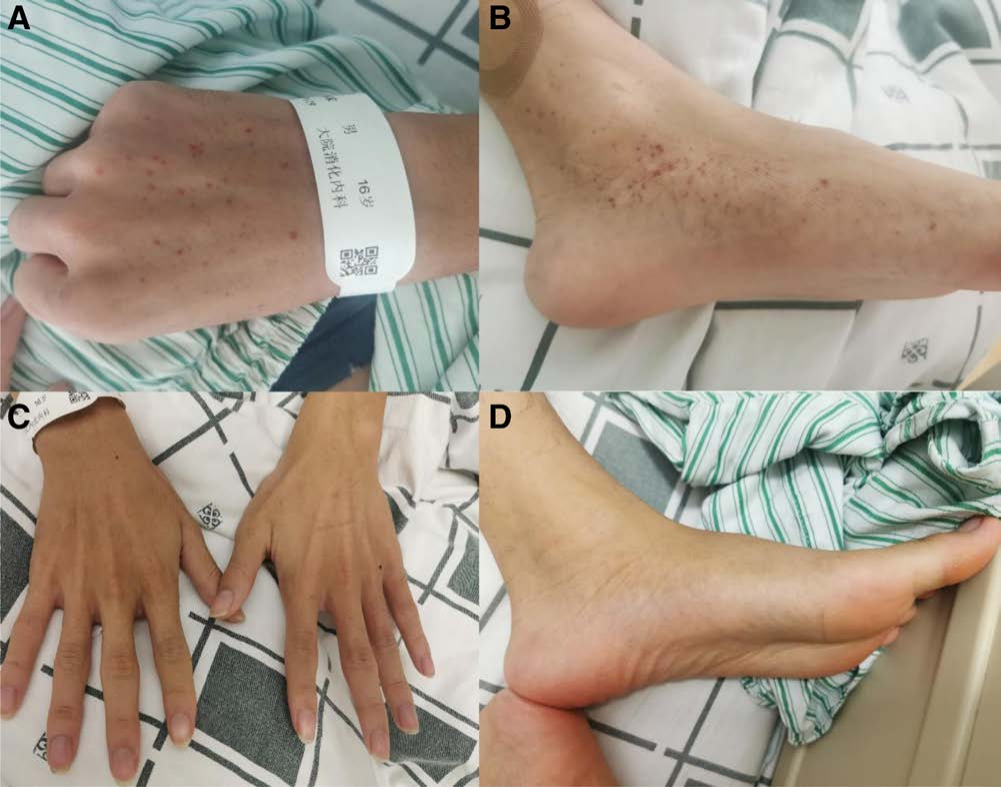
Pictures of petechiae. (A and B) Before treatment. (C and D) After treatment.

On the 8th day of admission, the patient developed scattered petechiae, primarily affecting the bilateral wrist and ankle joints, along with arthralgia (Fig. 3A and B). At this stage, the patient was diagnosed with mixed-type HSP. Subsequently, the patient was initiated on intravenous methylprednisolone sodium succinate at a dose of 1 mg/kg/d for 3 days. However, there was a progressive worsening of the scattered petechiae and arthralgia. Consequently, the treatment plan was escalated to include pulsed intravenous methylprednisolone sodium succinate (10 mg/kg/d) and intravenous gammaglobulin (0.1 g/kg/d). Notably, the patient’s petechiae, arthralgia, and abdominal pain eased after 2 days and resolved after 4 days (Fig. 3C and D). Subsequently, the patient was discharged from the hospital and prescribed oral methylprednisolone 32 mg/d for 2 weeks. During the 6-month follow-up period post-discharge, the patient attended 2 follow-up visits and reported no discomfort. Additionally, the follow-up renal function test revealed no abnormalities.

## 3. Discussion

### 3.1. Delayed diagnosis of Henoch–Schönlein purpura

In 2019, the Single Hub and Access point for pediatric Rheumatology in Europe Initiative established the diagnostic criteria for HSP, emphasizing the presence of purpura or petechiae predominantly on the lower limb with 1 of 4 conditions: (1) abdominal pain; (2) histopathology (IgA); (3) arthritis or arthralgia; and (4) renal involvement.^[8]^ Purpura or petechia serve as essential markers for diagnosis. Nonetheless, approximately 25% of patients do not initially present with purpura as their primary symptom.^[9]^ Fourteen percent to 36% of HSP patients experience GI symptoms before the appearance of purpura, with a time interval of 2 to 10 days between the onset of abdominal pain and the appearance of purpura.^[5,7,10]^ In practical settings, patients primarily exhibiting GI symptoms are often admitted to the gastroenterology departments, where gastroenterologists tend to prioritize differential diagnosis of acute and chronic GI diseases. Without palpable purpura, diagnosing HSP solely based on GI symptoms can be difficult, often leading to a delayed diagnosis. In this case, the patient was initially diagnosed with infectious gastroenteritis and intestinal obstruction, with a suspected diagnosis of Crohn disease. The definitive diagnosis of HSP was only confirmed after the emergence of petechiae and arthralgia.

The common GI manifestations of HSP are abdominal pain, nausea, and vomiting. Occasionally, it can progress to severe conditions such as intussusception, mesenteric ischemia, intestinal perforation, and GI bleeding.^[10–15]^ Intestinal obstruction is a rare complication in HSP, affecting only 9% of patients.^[16]^ The obstruction typically occurs in the small intestine.^[17–19]^ Endoscopic findings of HSP include mucosal erythema, edema, multiple irregular ulcers, and nodular changes.^[20]^ These lesions are likely associated with mucosal ischemia resulting from vasculitis. In the upper GI tract, the duodenum is the most commonly affected site, with the descending portion being more frequently affected than the bulb.^[21,22]^ In the lower GI tract, the terminal ileum is the most frequently affected site.^[21]^ Notably, esophageal lesions are rare, with a study reporting esophageal involvement in only 1 of 57 patients with HSP.^[22]^

The CT imaging characteristics of HSP patients are multifocal bowel wall thickening with segmental skip areas, primarily involving the jejunum and ileum.^[23]^ In this case, endoscopic observations revealed ulcerations in the duodenum, terminal ileum, and esophagus, presenting as irregular or annular shapes. Given the observation of pebble-like lesions in the terminal ileum, segmental thickening of the small intestine, combined with the patient’s age and arthralgia symptoms, a differential diagnosis of Crohn disease is warranted. IgA deposition in the capillary wall is thought to be characteristic of HSP. In contrast, Crohn disease lacks IgA deposition. Unfortunately, IgA staining is not routinely included in pathological examinations of the GI tract. This often results in missed opportunities for early HSP diagnosis. However, HSP may coexist with Crohn disease,^[24–26]^ which further complicates the differential diagnosis.

Notably, the abnormalities in coagulation function, immunoglobulin M, and IgA at admission may suggest markers for HSP. Elevated serum IgA levels have been reported in 50% to 70% of patients with HSP, but these elevations are not specific.^[27]^ Considering that the patient had undergone subtotal thyroidectomy for thyroid cancer 3 weeks earlier, the abnormalities in the aforementioned markers may also be attributed to a postoperative hypercoagulable state and coagulation factor deficiency. At present, there is a paucity of research on the efficacy of biomarkers for the early diagnosis of abdominal or mixed-type HSP, thereby limiting our comprehension of their potential diagnostic value.

### 3.2. Treatment for HSP patients with refractory GI symptoms

With regard to the treatment for HSP, the Single Hub and Access point for pediatric Rheumatology in Europe Initiative recommended that CS treatment could be considered for patients with severe abdominal pain and/or rectal bleeding.^[8]^ Specifically, the recommended oral CS dosage is prednisolone at 1 to 2 mg/kg/d for 1 to 2 weeks, followed by a gradual reduction over the subsequent 2 weeks. In cases of severe involvement, such as cerebral, pulmonary, or GI manifestations, pulsed intravenous methylprednisolone at 10 to 30 mg/kg, with a maximum dose of 1 g/d for 3 consecutive days, is recommended. Several studies have demonstrated that CS can reduce the intensity and duration of abdominal pain and arthralgia in the early stages of HSP.^[28,29]^

However, despite the use of CS, a significant proportion of patients with HSP continue to experience persistent GI symptoms.^[30]^ Refractory GI involvement is defined as the persistent GI symptoms that do not resolve within 3 days of glucocorticoid treatment (prednisone 1–2 mg/kg/d orally, maximum dose of 60–80 mg/d or equivalent doses of parenteral dexamethasone) or dependent (relapsing twice when glucocorticoid was tapered).^[31]^ At this point, IVIG is considered as a second-line treatment option.^[32]^ IVIG is a blood product composed of normal human immunoglobulin from thousands of healthy donors, primarily containing IgG and smaller amounts of IgA and IgM. IVIG functions as an immune modulator, regulating the activity of various cells within the innate and adaptive immune systems.^[31]^ Currently, there is limited research on the application of IVIG in HSP patients with severe GI involvement. However, studies have shown that patients with severe GI involvement who fail to respond to oral or intravenous CS (1–2 mg/kg/day) demonstrate improved outcomes when treated with a combination of IVIG.^[31,33,34]^ Öner et al reported that patients with GI involvement who failed to respond to pulse CS (30 mg/kg/d) attained complete remission following the combination of IVIG.^[35]^ These studies collectively support the effectiveness of IVIG in patients with refractory GI symptoms. In another perspective, the optimal timing of IVIG administration remains a subject of debate. Kang et al reported a case of a 15-year-old girl who failed to respond to a 1-month regimen of oral CS (1–2 mg/kg/day) combined with IVIG.^[36]^ Subsequently, after being treated with pulse CS (30 mg/kg/day) in combination with IVIG, her abdominal pain notably improved. Does the effectiveness of IVIG vary between the low-dose CS phase and the high-dose CS phase? Furthermore, the dosages of IVIG administered in the aforementioned studies varied from 0.4 to 2 g/kg/d. Does the dosage of IVIG influence its therapeutic efficacy? In this case, after the ineffectiveness of low-dose CS (1 mg/kg/d), the treatment regimen was adjusted to pulsed CS (10 mg/kg/d) combined with low-dose IVIG (0.1 g/kg/d). After 4 days, all symptoms had resolved. The patient tolerated the treatment well, experiencing no serious adverse events, such as anaphylaxis, hemodynamic shock, respiratory, or renal failure. Our report provides clinicians with a valuable reference for managing HSP patients with refractory GI symptoms. However, this is a single-case report and lacks a control group. Future high-quality comparative trials are crucial to confirm the efficacy of IVIG.

### 3.3. Limitations

Histopathology is regarded as one of the optional diagnostic criteria. Unfortunately, IgA staining was not performed during the endoscopic biopsy, preventing confirmation of the HSP diagnosis at the pathological level. Furthermore, despite our desire to gain a more profound understanding of the patient’s GI status posttreatment through imaging assessments, the patient refused the request for a follow-up examination. This was due to their significantly improved symptoms, as well as the high costs and potential discomfort associated with endoscopy and computed tomography enterography. Consequently, this decision hindered our ability to further comprehend the patient’s GI condition posttreatment at the imaging level.

## 4. Conclusion

The diagnosis of HSP can be challenging for patients exhibiting complex GI manifestations, particularly when characteristic purpura emerges following GI symptoms. Gastroenterologists should consider HSP as a potential differential diagnosis. For HSP patients with refractory GI symptoms, the combination of IVIG represents a treatment option worthy of consideration.

## Author contributions

**Conceptualization:** Liji Chen, Cailing Zhong.

**Data curation:** Ming Luo, Linkun Cai.

**Writing – original draft:** Liji Chen, Cailing Zhong, Longxiu Fan.

**Writing – review & editing:** Beiping Zhang, Haiyan Zhang.
